# A First-Trimester Serum Proteomic Signature for Early Prediction of Preeclampsia: Integrated Untargeted and Targeted Mass Spectrometry with Machine Learning

**DOI:** 10.3390/life16081264

**Published:** 2026-07-30

**Authors:** Natalia Starodubtseva, Alina Poluektova, Alisa Tokareva, Alexey Kononikhin, Alexander Brzhozovskiy, Anna Bugrova, Evgenii Kukaev, Zulfiya Khodzhaeva, Evgeny Nikolaev, Gennady Sukhikh

**Affiliations:** 1 V.I. Kulakov National Medical Research Center for Obstetrics Gynecology and Perinatology, Ministry of Healthcare of Russian Federation, 117997 Moscow, Russia; 2 Moscow Center for Advanced Studies, 123592 Moscow, Russia; 3 The Center for Bio- and Medical Technologies, 121205 Moscow, Russia; 4 Emanuel Institute of Biochemical Physics, Russian Academy of Sciences, 119334 Moscow, Russia; 5 V.L. Talrose Institute for Energy Problems of Chemical Physics, N.N. Semenov Federal Research Center for Chemical Physics, Russian Academy of Sciences, 119334 Moscow, Russia; 6 Department of Obstetrics, Gynecology, Perinatology and Reproductology, Institute of Professional Education, Federal State Autonomous Educational Institution of Higher Education I.M. Sechenov First Moscow State Medical University of the Ministry of Health of the Russian Federation, 119991 Moscow, Russia

**Keywords:** preeclampsia, first-trimester screening, serum proteomics, prognosis, DIA-MS, MRM-MS, machine learning, biomarkers, complement system, mass spectrometry

## Abstract

First-trimester prediction of preeclampsia (PE) remains a major clinical challenge, particularly outside specialized fetal medicine centers. This study aimed to identify and validate serum protein biomarkers for early PE prediction using an integrated proteomic approach. A prospective cohort of 64 first-trimester singleton pregnancies (32 future PE cases, 32 matched controls) was analyzed. Untargeted proteomics was performed using DIA-PASEF-MS, followed by targeted cross-platform verification with MRM-MS. Machine learning classifiers (support vector machines, SVM, and random forest) were trained on differentially abundant proteins (FDR < 0.01, VIP > 1.5). DIA-MS identified 33 protein markers associated with complement activation, IGF transport regulation, and platelet degranulation. An SVM model with a linear kernel achieved 95% accuracy (AUC = 0.95, sensitivity = 95%, specificity = 97%). Four markers (AFM, AHSG, C8A, IGHG1) were confirmed across platforms, confirming the discovery findings. Cross-platform correlation was high: 71% of overlapping proteins showed r > 0.5 (*p* < 0.001), with the highest concordance observed for potential PE marker AHSG (r = 0.8, *p* < 0.001). PRSS1, IGHV1-4, and SERPINC1 showed a strong correlation with proteinuria (|r| > 0.5, *p* < 0.05), linking the proteomic signature to clinical severity. Integrated DIA-MS and MRM-MS proteomics yields a reproducible, high-performance serum signature for first-trimester PE prediction. The identified markers reflect core pathophysiological pathways and offer potential to augment current FMF-based screening algorithms.

## 1. Introduction

Preeclampsia (PE) is a leading cause of maternal and perinatal morbidity and mortality globally, particularly when it develops early and leads to indicated preterm delivery [1]. Consequently, the reliable first-trimester identification of high-risk women is a paramount objective in modern obstetrics. This remains challenging, especially for nulliparous patients, in whom traditional risk-factor-based screening performs suboptimally [2,3]. Current guidelines rely primarily on maternal demographic and historical factors, an approach that limits detection rates and leaves a substantial proportion of cases undetected [3,4,5,6,7,8,9,10].

While more sophisticated first-trimester algorithms, such as the Fetal Medicine Foundation (FMF) model—which integrates maternal factors with uterine artery Doppler and biochemical markers—can detect 75–90% of early or preterm preeclampsia at a 10–16% false-positive rate, their performance is imperfect and varies across populations and settings [3,4,11,12]. Furthermore, the implementation of these multivariable tools requires standardized measurements, specialized software, and trained personnel [7,13,14]. Critically, they may not fully capture the complex systemic pathophysiological shifts that precede clinical disease [15,16]. Maternal serum offers a compelling matrix for biomarker discovery due to its minimally invasive collection and its reflection of systemic processes underpinning preeclampsia, including placental dysfunction, endothelial activation, and inflammation [15].

Modern mass-spectrometry-based proteomics enables the specific, reproducible, and multiplexed quantification of hundreds of serum proteins, often surpassing conventional immunoassays in analytical performance [15,17,18,19,20,21]. Data-Independent Acquisition—Parallel Accumulation Serial Fragmentation (DIA-PASEF) provides unbiased, broad-coverage discovery of the serum proteome, while targeted multiple reaction monitoring (MRM) mass spectrometry permits precise validation of candidate proteins across cohorts, establishing a powerful complementary pipeline [15,18,19,20,21,22,23]. The integration of these advanced proteomic approaches in early pregnancy holds promise for uncovering molecular signatures of subsequent preeclampsia, refining individual risk stratification beyond existing algorithms, and informing more effective preventive strategies [16,24,25].

This study employs an integrated DIA-PASEF-MS and targeted MRM-MS strategy on first-trimester serum samples from women who later developed preeclampsia and matched controls. The objective is to identify robust protein biomarkers and to construct predictive models with the potential to augment current FMF-based screening in routine clinical practice.

## 2. Materials and Methods

### 2.1. Study Design

This prospective cohort study was conducted at the I. Kulakov National Medical Research Center for Obstetrics, Gynecology, and Perinatology (Moscow, Russia) from January to December 2022. The analytical cohort was selected from an initial screening population of 1869 women aged 18–45 years who underwent routine first-trimester FMF-based screening between 11^+2^ and 14^+2^ weeks of gestation. The final cohort comprised 64 women with singleton pregnancies, including 32 who subsequently developed PE and 32 age-matched controls with uncomplicated pregnancies. Exclusion criteria were pre-existing diabetes mellitus, autoimmune disease, a history of solid-organ transplantation, known malignancy, or fetal chromosomal abnormalities. 

Preeclampsia was defined according to contemporary guidelines as de novo hypertension after 20 weeks of gestation accompanied by proteinuria, maternal organ dysfunction (renal, hepatic, hematologic, or neurologic), or uteroplacental dysfunction including fetal growth restriction [13]. The control group consisted of age-matched (*p* > 0.05) women with uncomplicated singleton pregnancies who conceived spontaneously and did not develop any hypertensive disorder, gestational diabetes, preterm delivery before 37 weeks, or intrauterine growth restriction (estimated fetal weight < 10th percentile) and did not have high FMF-based first-trimester risk of PE.

Written informed consent was obtained from all participants at enrollment. A standardized first-trimester assessment included measurement of maternal weight, height, and blood pressure [13], along with collection of detailed obstetric and medical history. Ultrasonographic evaluation involved transabdominal color Doppler assessment of the uterine arteries with calculation of the pulsatility index (UtA-PI) [14]. 

Venous blood was collected into Serum Z/9 tubes (Monovette, Sarstedt, Germany) and processed within 2 h. After centrifugation at 300× *g* for 20 min at room temperature, the serum supernatant was aliquoted and stored at −80 °C until proteomic analysis. All samples underwent one freeze–thaw cycle immediately before sample preparation.

Serum concentrations of placental growth factor (PlGF) and pregnancy-associated plasma protein-A (PAPP-A) were measured using the Delfia Xpress system (PerkinElmer, Shelton, CT, USA) as per the manufacturer’s instructions. Residual serum was aliquoted and stored at −80 °C for subsequent untargeted DIA-MS and targeted MRM-MS proteomic analyses of first-trimester biomarkers [22]. The study protocol was approved by the Institutional Review Board of the I. Kulakov Center (protocol No. 2, 9 March 2017). All procedures followed the principles of the Declaration of Helsinki and Good Clinical Practice guidelines.

### 2.2. Serum Preparation

Serum samples and a surrogate matrix of bovine serum albumin (BSA, 10 mg/mL in PBS) were subjected to tryptic digestion following standard procedures [20,22,23,26]. Briefly, 10 µL of serum was diluted in denaturation buffer containing 7.2 M urea, 16 mM dithiothreitol, and 240 mM Tris-HCl (pH 8.0) and incubated for 30 min at 37 °C. Alkylation was performed with iodoacetamide at a final concentration of 40 mM for 30 min at room temperature in the dark. Trypsin (Trypsin Gold, Promega, Madison, WI, USA) was added at a 1:25 enzyme-to-protein ratio, and digestion proceeded overnight at 37 °C. Total protein concentration was determined using a bicinchoninic acid (BCA) assay kit (Thermo Fisher Scientific, Waltham, MA, USA). Digestion was terminated by acidification with 1.0% formic acid to pH ≤ 2. The resulting peptide mixture was adjusted to 1 µg/µL, kept on ice, and analyzed by mass spectrometry on the same day.

For targeted MRM-MS analysis of 139 serum proteins, an in-house panel based on the BAK125/270 MRM kit (MRM Proteomics, Montreal, QC, Canada) was employed. The rationale for selecting this panel was two-fold: (1) its broad coverage of pathways relevant to systemic inflammatory and vascular dysfunction [20,21,22], which are key to PE pathogenesis, and (2) its ready availability with pre-validated stable isotope-labeled internal standard (SIS) peptides, allowing rapid quantitative orthogonal validation of candidate markers without the need for de novo assay development. This strategy enabled us to efficiently cross-validate the DIA-MS discovery findings.

SIS peptides and corresponding natural synthetic proteotypic peptides (NATs) were synthesized and characterized at the Omics Laboratory of Skolkovo Institute of Science and Technology (Skoltech, Moscow, Russia) [20,21]. The SIS peptide panel featured isotopically labeled C-terminal lysine (+8 Da) or arginine (+10 Da) residues. 

The SIS peptide mixture was dissolved and diluted to a working solution at 10× the lower limit of quantification (LLOQ). For each sample, 40 µL of serum digest was combined with 10 µL of the SIS peptide mixture. Peptide purification was performed via solid-phase extraction using Oasis HLB 96-well plates. After conditioning and sample loading, wells were washed and peptides were eluted with 70% acetonitrile/0.1% formic acid. Eluates were dried in a vacuum concentrator and stored at −80 °C until analysis.

### 2.3. Targeted HPLC-MS Analysis

For targeted high-performance liquid chromatography–tandem mass spectrometry (HPLC-MRM-MS) analysis, dried peptide samples were reconstituted in 0.1% formic acid to a final concentration of 1 µg/µL. Aliquots of 10 µL from each reconstituted serum digest, quality control (QC) samples, and calibration standards were injected onto a Zorbax Eclipse Plus reversed-phase UHPLC column (Agilent Technologies, Santa Clara, CA, USA) connected to an ExionLC™ system interfaced with a SCIEX QTRAP 6500+ mass spectrometer (SCIEX, Toronto, ON, Canada). Peptides were separated at a flow rate of 0.4 mL/min over a 60 min multistep gradient [22]. 

For calibration standards and QC samples, 40 µL of surrogate BSA digest was mixed with 10 µL of SIS mixture and 10 µL of NAT mixture. A lyophilized mixture of NAT peptides, pre-balanced at the LLOQ for each analyte, was dissolved and serially diluted to generate eight calibration levels: 100×, 40×, 16×, 4×, 2×, 0.5×, 0.25× and 0.1× LLOQ. QC samples at three concentration levels (0.35× LLOQ, QC-A; 3.5× LLOQ, QC-B; and 35× LLOQ, QC-C) were analyzed in triplicate. 

MRM data were processed using Skyline software [27]. Quantification adhered to ICH guidelines for Bioanalytical Method Validation [28], employing weighted (1/x^2^) linear regression of SIS-to-native peak area ratios. Calibration curve performance was assessed in Skyline, with accuracy considered acceptable when measured concentrations for at least 6 of 9 calibration points and at least 1 of 3 QC replicates at each level were within ±20% of nominal values.

### 2.4. Non-Targeted HPLC-DIA-PASEF-MS Analysis

Tryptic peptide fractions were analyzed on a Dionex Ultimate 3000 nano-LC system (Thermo Fisher Scientific, Waltham, MA, USA) coupled with a timsTOF Pro mass spectrometer (Bruker Daltonics, Billerica, MA, USA). A total of 1 µL of each peptide sample was injected onto a packed emitter C18 column (25 cm × 75 µm, 1.6 µm; Ion Optics, Parkville, Australia). LC separation was carried out at a flow rate of 400 nl/min using a 90 min linear gradient from 2% to 37% of solvent B (0.1% FA in ACN), followed by a column wash step (10 min isocratic elution with 90% solvent B) and equilibration (15 min, isocratic elution with 2% solvent B). QC samples were injected periodically throughout the analytical sequence to monitor instrument performance and retention time stability.

MS data were acquired using the DIA-PASEF method. The electrospray ionization (ESI) source parameters were set as follows: capillary voltage 1400 V, dry gas flow—3.0 L/min at 180 °C. MS and MS/MS spectra were recorded in the range of 100 to 1700 m/z and in the ion mobility range from 0.6 to 1.6 V·s/cm^2^. The scan time (ramp) was set to 100 ms. Collision energy changed linearly depending on mobility: from 59 eV at 1/K0 = 1.6 V·s/cm^2^ to 20 eV at 1/K0 = 0.6 V·s/cm^2^.

The LC-MS/MS spectra were analyzed using the DIA NN software (Data-Independent Acquisition by Neural Networks, version 1.8.1) in library-free mode with the following parameters: mass accuracy—20 ppm; MS1 accuracy—20 ppm; and peptide length range—from 7 to 30 amino acids [7,8,9]. The search was performed against the SwissProt Human database with carbamidomethylation (C) and oxidation (M) specified as variable modifications. The false discovery rate threshold was set at 0.1%. Data filtering and calculation of LFQ values were performed using the R package DIAgui [29].

### 2.5. Statistical Analysis

Continuous clinical variables were summarized as medians (first quartile; third quartile) and categorical variables as counts (percentage). Between-group differences were assessed using the Mann–Whitney U test for continuous variables and Pearson’s chi-square test for categorical variables, with statistical significance defined as *p* < 0.05. Fisher’s exact test was used for all 2 × 2 contingency tables where any cell contained a count of zero.

For untargeted DIA-MS data, only proteins detected in ≥70% of samples were analyzed. Missing values were imputed in Perseus (MaxQuant environment) using a normal distribution-based procedure [30]. 

For targeted MRM-MS data to correct for batch effects and analytical variability, data normalization was performed using RobNorm methods [31]. For each protein, the mean CV was calculated across standard and quality control samples between batches; proteins with a CV exceeding 50% were excluded from further analysis.

Significant proteins from both targeted and untargeted datasets were identified using the Mann–Whitney U test with Benjamini–Hochberg correction (false discovery rate (FDR)-adjusted *p*-value < 0.01). Also, proteins from the untargeted dataset were compared between subgroups with early (*n* = 8) and late (*n* = 24) PE by the same procedure. Principal component analysis (PCA) and orthogonal projections to latent structures discriminant analysis (OPLS-DA) with standard scaling were performed on each proteomic profile, and features with variable importance in projection (VIP) > 1.5 and FDR < 0.01 were retained as protein markers [32]. The DIA protein markers were used for building various machine learning models: OPLS-DA, support vector machines (SVMs) with linear, polynomial, radial and sigmoid kernels and random forest models [33,34]. Hyperparameters of SVM with polynomial (degree, γ and free coefficients), radial (γ-coefficient) and sigmoid kernels (γ and free coefficients) were optimized using particle swarm optimization (PSO) with 5-fold cross-validation by lab-created scripts [35], and the number of trees in random forest models was 3000. Model performance was evaluated by 10-fold cross-validation which calculated sensitivity, specificity, accuracy, area under the receiver-operating characteristic curve (AUC), positive predictive value (PPV), negative predictive value (NPV) and F-score. 

To evaluate the incremental predictive value of the proteomic signature, a second set of models was constructed by integrating the protein markers with clinical and anamnestic parameters. These included maternal age, body mass index (BMI), history of preeclampsia, in vitro fertilization (IVF), habitual miscarriage, fetal sex, nulliparity, obesity status, and first-trimester screening variables—specifically, multiples of the median (MoM) values for pregnancy-associated plasma protein-A (PAPP-A), placental growth factor (PlGF), and free β-human chorionic gonadotropin (free β-hCG), as well as mean arterial pressure (MAP) and uterine artery pulsatility index (UtA-PI). The performance of the proteome-based and combined proteome–clinical models was compared against the FMF screening results using a comprehensive set of metrics, including AUC, accuracy, sensitivity, specificity, expected calibration error (ECE), decision curve analysis, and net reclassification improvement (NRI).

Pathway enrichment analysis was conducted using STRING (FDR < 0.01) [36] to identify biological processes and molecular pathways associated with the discovered protein markers.

Cross-platform concordance between DIA-MS and MRM-MS protein measurements was assessed using the Pearson test. Associations between DIA-MS protein levels and continuous clinical parameters were evaluated using the Spearman correlation test. A threshold of statistical significance was set at *p* < 0.05 for all analyses.

Linear mixed models were employed to assess whether baseline clinical parameters, showing statistically significant differences between PE and control groups, exerted confounding effects on protein marker levels. Each protein marker was modeled as the dependent variable, with clinical parameters (nulliparity, previous PE, habitual miscarriage, previous preterm delivery, etc.) included as fixed effects. A clinical parameter was considered to have a statistically significant confounding effect on protein levels when the corresponding *p*-value was < 0.05.

Statistical analysis was performed by scripts based on R version 4.3.2 [37] and the RStudio environment (version 2023.09.1) [38]. A comprehensive suite of R packages was employed to support both statistical modeling and data visualization. For modeling and machine learning tasks, the packages ropls 1.34.0 [39], effsize 0.8.1 [40], pwr 1.3-0 [41], e1071 1.7-16 [42], caret 7.0-1 [43], dplyr 1.1.4 [44], RandomForest [34], lme4 2.0-1 [45], pbkrtest 0.5.5 [46], CalibratR 0.1.2 [47] and nricens 1.6 [48] were utilized. Visualization of the results was accomplished using ggplot2 3.5.2 [49], reshape2 1.4.4 [50], forcats 1.0.0 [51], ggrepel 0.9.6 [52], pheatmap 1.013 [53] and pROC 1.18.5 [54] and gmish 0.10 [48].

## 3. Results

### 3.1. Clinical Characteristics

The final cohort comprised 32 women who subsequently developed PE and 32 age-matched controls with uncomplicated pregnancies. The clinical characteristics of both groups are summarized in Table 1.

Maternal age, body mass index (BMI), gestational age at sample collection, mean arterial pressure (MAP) MoM, and the use of assisted reproductive technologies (IVF) did not differ significantly between the groups (all *p* > 0.05). However, women who later developed PE were more frequently nulliparous (72% vs. 34%, *p* < 0.001), had a higher prevalence of previous PE (22% vs. 0%, *p* = 0.02), habitual miscarriage (38% vs. 0%, *p* < 0.001), and previous preterm delivery (25% vs. 0%, *p* = 0.01) compared to controls.

First-trimester FMF-based screening identified a high risk for PE in 53% of women who subsequently developed the disease, whereas none of the controls were classified as high risk (*p* < 0.001). First-trimester placental growth factor (PlGF) levels (MoM) were significantly lower in the PE group compared to controls (median 0.55 vs. 0.74, *p* = 0.002).

Regarding pregnancy outcomes, women in the PE group delivered earlier than controls (median 37.2 vs. 39.4 weeks, *p* < 0.001) and had higher maximum systolic and diastolic blood pressures (135 vs. 115 mmHg and 89 vs. 70 mmHg, respectively, both *p* < 0.001). The PE group also demonstrated higher umbilical artery pulsatility index (0.95 vs. 0.79, *p* = 0.002) and lower cerebroplacental ratio (1.44 vs. 1.89, *p* < 0.001).

Markers of disease severity were significantly elevated in the PE group, including 24 h proteinuria (1.1 vs. 0 g/L, *p* < 0.001) and serum creatinine (80.8 vs. 66.6 µM/L, *p* < 0.001). Intrapartum blood loss was higher in the PE group (700 vs. 300 mL, *p* < 0.001), and emergency cesarean section was more frequent (59% vs. 3%, *p* < 0.001). Neonatal outcomes were less favorable in the PE group, with lower birthweight (2745 vs. 3400 g, *p* < 0.001) and lower Apgar scores at both 1 min (*p* = 0.002) and 5 min (*p* = 0.001) compared to controls.

### 3.2. Non-Targeted DIA-MS Proteome Profile

**Figure 1:**
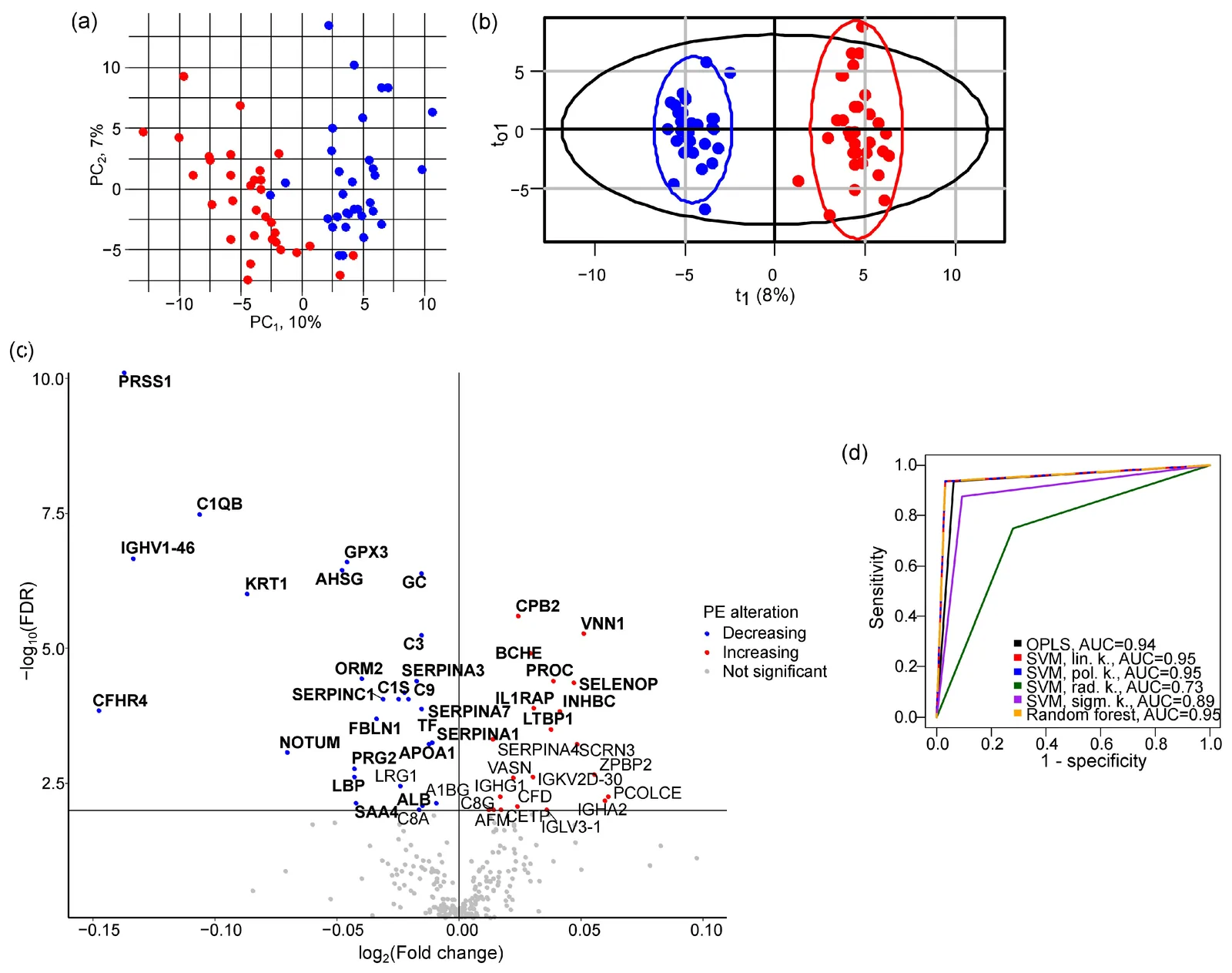
Overview of serum proteomic data analysis and model performance for first-trimester prediction of PE using DIA-MS. (**a**) PCA score plot showing sample distribution in principal component space. Red circles represent samples from patients with PE (*n* = 32), while blue circles represent samples from the control group (*n* = 32). (**b**) OPLS-DA score plot illustrating group separation between PE patients (red) and controls (blue). The model effectively distinguishes the two groups based on first-trimester serum protein profiles. (**c**) Volcano plot of all proteins quantified by DIA-MS. The x-axis represents the log_2_(fold change), calculated as the ratio of the median protein level in the PE group to the median level in the control group. The y-axis shows the −log_10_(FDR-adjusted *p*-value). Proteins with FDR < 0.01 are labeled in regular text; proteins meeting both FDR < 0.01 and VIP > 1.5 are highlighted in bold text. (**d**) Receiver operating characteristic (ROC) curves generated from 10-fold cross-validation of machine learning models. Each curve represents the diagnostic performance of a classifier in distinguishing future PE patients from controls. Corresponding AUC values are reported.

Non-targeted HPLC-DIA-MS analysis of first-trimester serum identified over 455 protein groups. Statistical analysis was performed on 274 proteins consistently detected in at least 70% of samples (Table S1). PCA revealed clear separation between samples from women who later developed PE and controls. This separation was further supported by an OPLS-DA model demonstrating high explanatory and predictive capability (R2Y = 0.96, Q2Y = 0.78) (Figure 1a,b).

Differential abundance analysis identified 69 proteins with significant unadjusted *p*-values (*p* < 0.01), of which 48 remained significant following FDR correction (FDR < 0.01). From this set, 33 proteins with a VIP score exceeding 1.5 were designated as candidate PE markers (Figure 1c, Table S1). The majority of these proteins were downregulated in serum from women who later developed PE (CFHR4, PRSS1, IGHV1-46, C1QB, KRT1, NOTUM, AHSG, GPX3, LBP, PRG2, SAA4, ORM2, FBLN1, SERPINC1, SERPINA7, C1S, C9, SERPINA3, C3, TF, GC, ALB, APOA1 and SERPINA1), while a smaller subset was upregulated (CFD, CPB2, BCHE, IL1RAP, LTBP1, PROC, INHBC, SELENOP and VNN1).

When these 33 DIA-derived markers were used as input features for classification models, SVM with linear and polynomial kernels and a random forest classifier achieved the highest quality metrics (PPV and NPV of 97% and 95%, respectively) during 10-fold cross-validation (Table 2, Figure 1d). 

**Figure 2:**
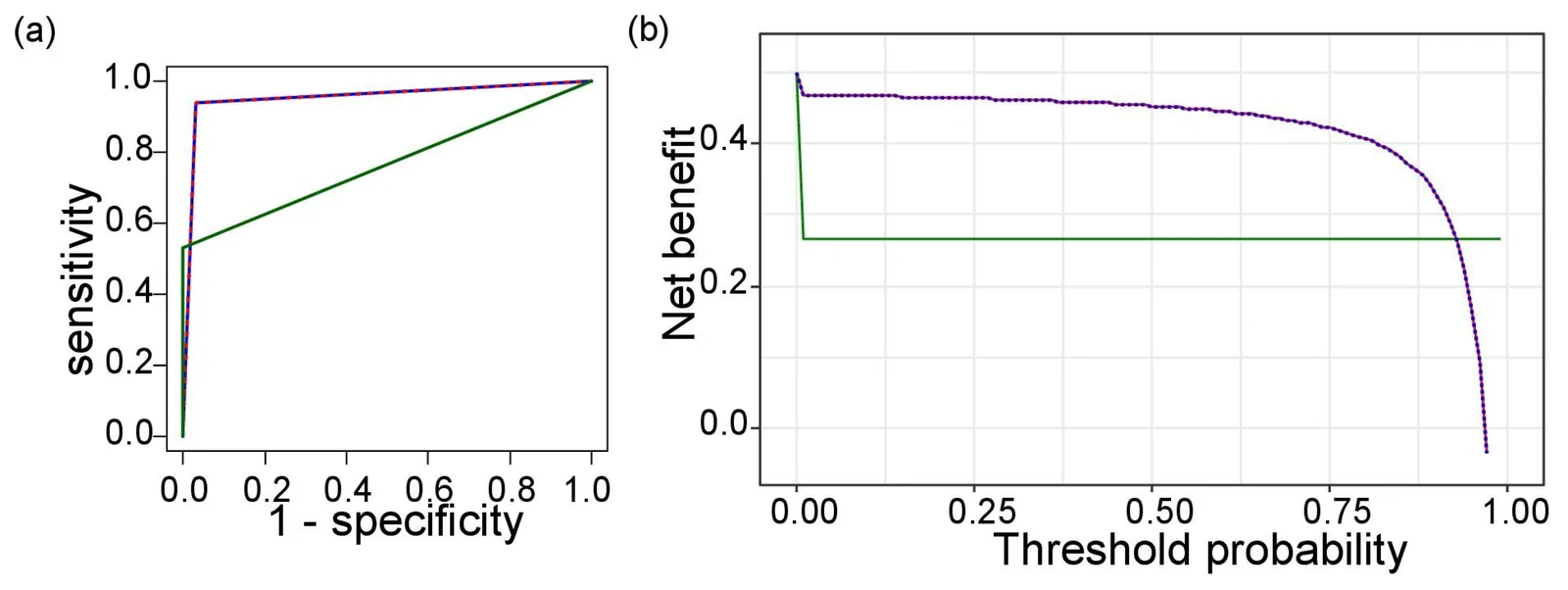
Comparison of predictive models for first-trimester prediction of PE. (**a**) ROC curves comparing the FMF-based screening model (green), the SVM model based on the 33-protein DIA-MS signature (red), and the combined proteomic and clinical model (blue). The proteomic models show superior AUC and sensitivity. (**b**) Decision curve analysis demonstrating the net benefit of the proteomic models across a range of threshold probabilities compared to the FMF screening model.

To assess the clinical utility of the proteomic signature, we compared its predictive performance against the established first-trimester FMF-based screening algorithm and evaluated the added value of integrating clinical and anamnestic parameters. The FMF screening model, applied to our cohort, classified 53% (17/32) of the women who later developed PE as high risk, with no controls classified as high risk, yielding an AUC of 0.77 (95% CI: 0.68–0.85). The SVM model based on the 33 protein markers significantly outperformed the FMF model, achieving an AUC of 0.95 (95% CI: 0.91–1.00) (Figure 2a). Its sensitivity was notably higher (94% vs. 53%) while maintaining a high specificity (97%) (Table 3).

When clinical and anamnestic parameters were added to the proteomic model, performance metrics remained unchanged (AUC = 0.95). This suggests that the proteomic signature already captures much of the predictive information contained within these conventional risk factors. The ECE for the proteomic models (0.047) was substantially lower than for the FMF model (0.24), indicating superior calibration between predicted probabilities and observed outcomes. Decision curve analysis further demonstrated that the proteomic models provided a greater net benefit across a wide range of clinically relevant threshold probabilities compared to the FMF screening strategy (Figure 2b).

The NRI index for the proteomic-included model compared to the FMF model was 0.38 (95% CI: 0.20–0.56, *p* < 0.001). This indicates that the proteomic model reclassifies a significant proportion of individuals into more appropriate risk categories. The most notable reclassifications occurred for FMF low-risk women who developed PE (correctly reclassified as high risk by the proteomic model) and FMF high-risk controls (correctly reclassified as low risk).

**Figure 3:**
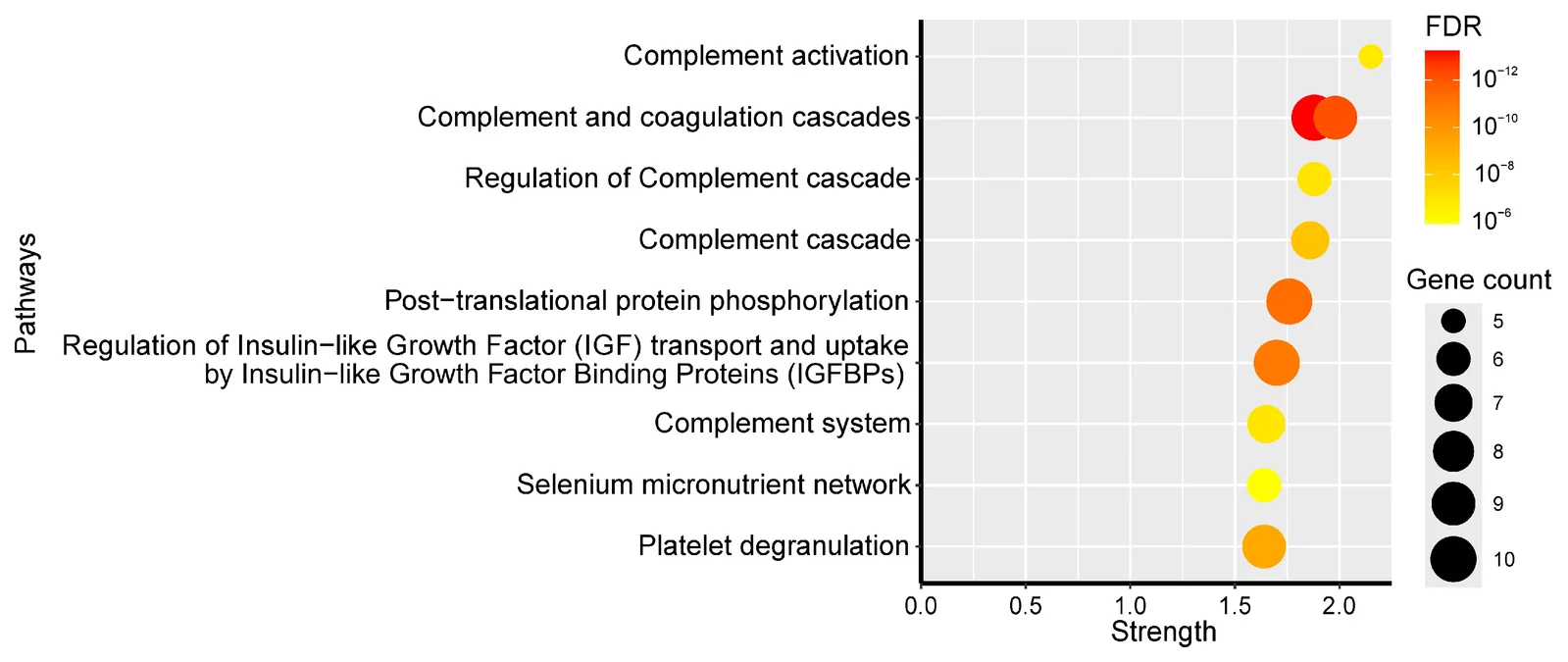
The 10 most enriched pathways among DIA-MS-detected protein markers of PE. Pathway enrichment analysis (STRING, FDR < 0.01) identified complement activation as the dominant pathway, along with significant enrichment of protein phosphorylation, IGF transport regulation, platelet degranulation, and selenium-related micronutrient networks.

Pathway enrichment analysis demonstrated that this protein set was strongly associated with complement activation pathways and also mapped to biological processes including post-translational protein phosphorylation, regulation of insulin-like growth factor (IGF) transport and uptake, platelet degranulation, and selenium-related micronutrient networks (Figure 3, Table S2).

**Figure 4:**
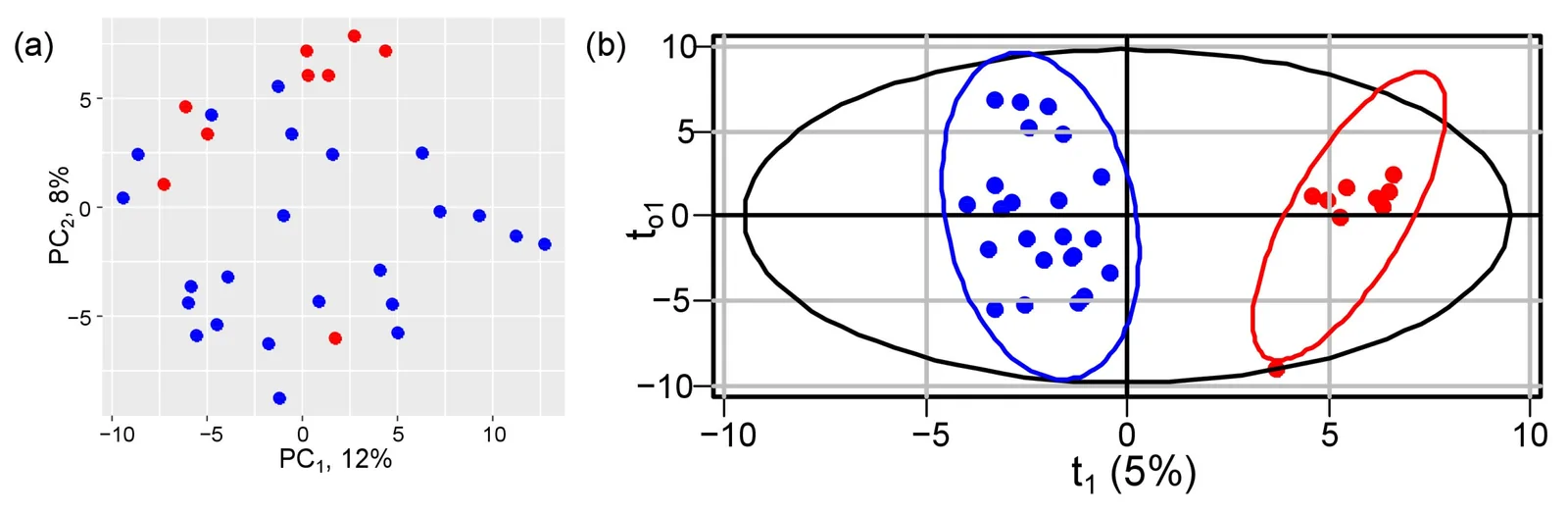
Proteomic profiling of early-onset versus late-onset PE. (**a**) PCA score plot showing the distribution of serum samples from women with early-onset PE (red, *n* = 7) and late-onset PE (blue, *n* = 25) in the first two principal components (PC1 and PC2). The substantial overlap between groups indicates limited proteomic discrimination based on disease timing. (**b**) OPLS-DA score plot illustrating the separation between early-onset and late-onset PE samples. The model exhibits limited predictive capacity (Q^2^Y = 0.18), likely attributable to the marked class imbalance (early-onset: *n* = 7; late-onset: *n* = 25) and the modest sample size available for subgroup analysis.

No statistically significant differences in first-trimester serum protein expression were observed between women who subsequently developed early-onset versus late-onset PE. PCA revealed poor separation between these two subgroups (Figure 4a). Consistent with this finding, an OPLS-DA model constructed to discriminate PE cases according to the timing of disease onset demonstrated limited explanatory power for the independent variables (R^2^X = 0.13), while showing high explanatory capacity for the dependent variable (R^2^Y = 0.93) but low predictive ability (Q^2^Y = 0.18) (Figure 4b). The marked discrepancy between explained and predicted variance (R^2^Y vs. Q^2^Y) is attributable to the substantial class imbalance between the late-onset (*n* = 25) and early-onset (*n* = 7) PE groups, with a ratio of approximately 3:1.

### 3.3. Targeted MRM-MS Cross-Platform Confirmation

**Figure 5:**
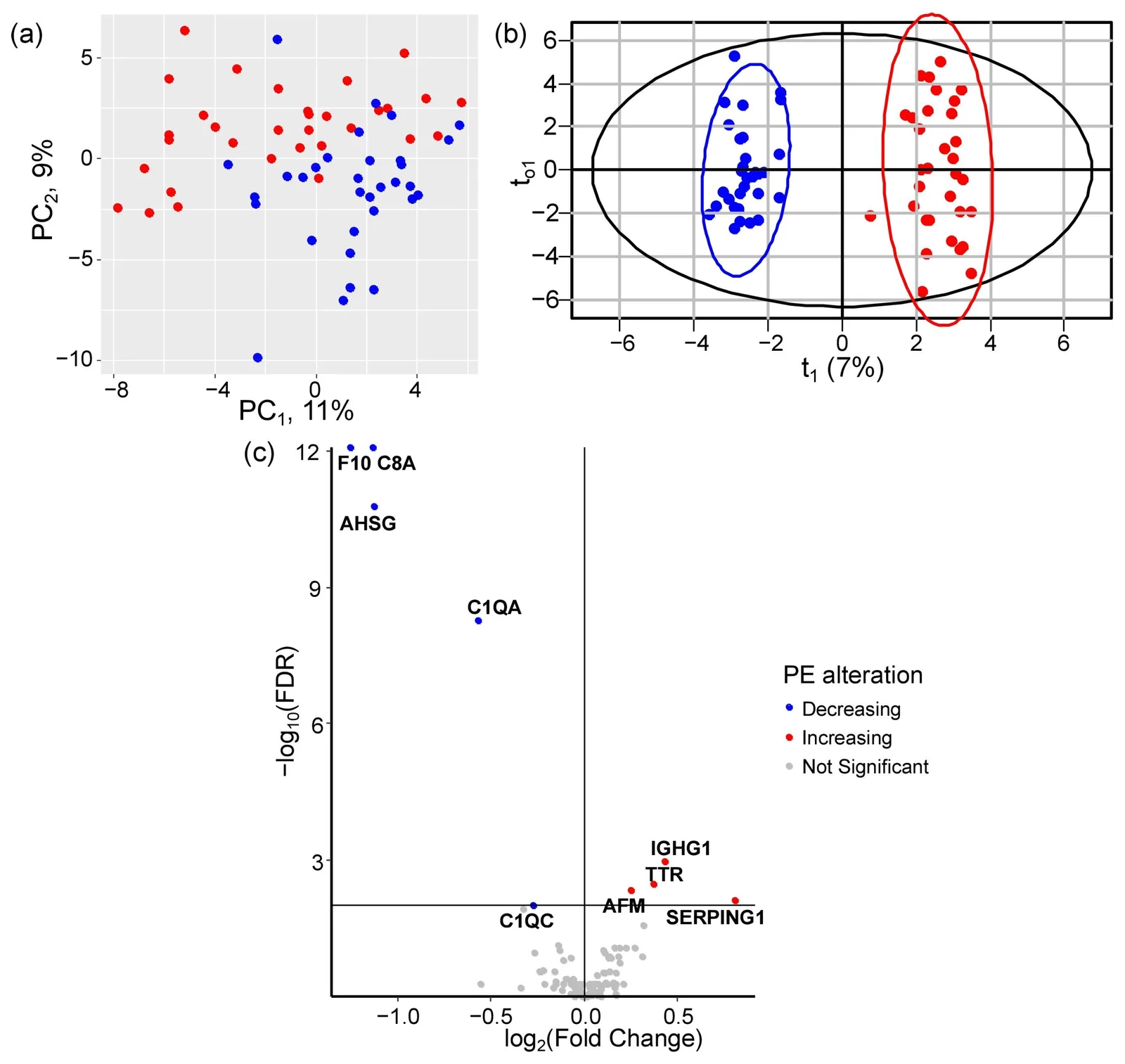
Overview of serum proteomic data analysis and predictive model performance for first-trimester prediction of PE using MRM-MS. (**a**) PCA of serum proteomic data. Samples are projected onto the first two principal components (PC1 and PC2) based on MRM-MS–derived proteomic profiles. Red dots represent patients who later developed PE (*n* = 32), and blue dots represent control subjects with uncomplicated pregnancies (*n* = 32). The PCA plot illustrates the natural variance and clustering tendency between the two groups prior to supervised modeling. (**b**) OPLS-DA score plot. Supervised classification of the same serum samples highlights the separation between future PE cases (red) and controls (blue). OPLS-DA maximizes covariance between proteomic data and group membership, enabling identification of latent variables driving group discrimination. (**c**) Volcano plot of individual proteins quantified by MRM-MS. The x-axis represents the log_2_(fold change), calculated as the ratio of the median protein level in the PE group to the median level in the control group. The y-axis shows the −log_10_(FDR-adjusted *p*-value). Among these, proteins with VIP scores > 1.5 (from OPLS-DA) are shown in bold, indicating strong discriminative and predictive potential for first-trimester PE risk assessment.

Targeted HPLC-MRM-MS quantification was successfully performed for 104 plasma proteins across all serum samples (*n* = 64) (Table S4). Unsupervised PCA again resolved two distinct clusters corresponding to future clinical outcomes. A subsequent OPLS-DA model based on the targeted panel demonstrated robust discriminatory power (R^2^Y = 0.94, Q^2^Y = 0.74) (Figure 5a,b).

Among the quantified proteins, 12 exhibited significant unadjusted *p*-values (*p* < 0.01), with nine proteins remaining significant after FDR correction (FDR < 0.01) and possessing high VIP scores (VIP > 1.5) in the OPLS-DA model (Table S3, Figure 5c). Notably, four of these nine verified proteins (AFM, AHSG, C8A, IGHG1) overlapped with candidates identified in the discovery phase, providing cross-platform confirmation.

**Figure 6:**
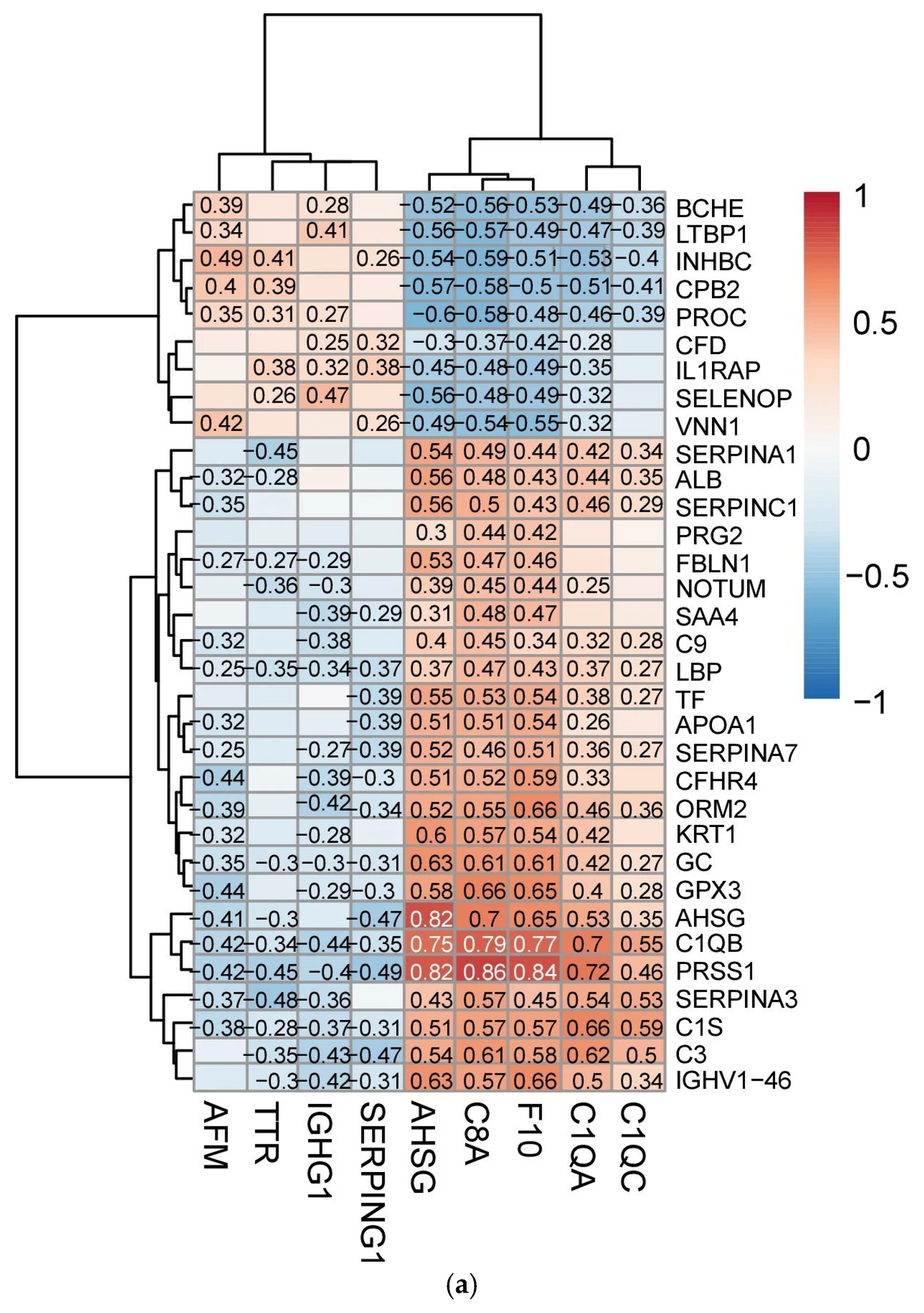

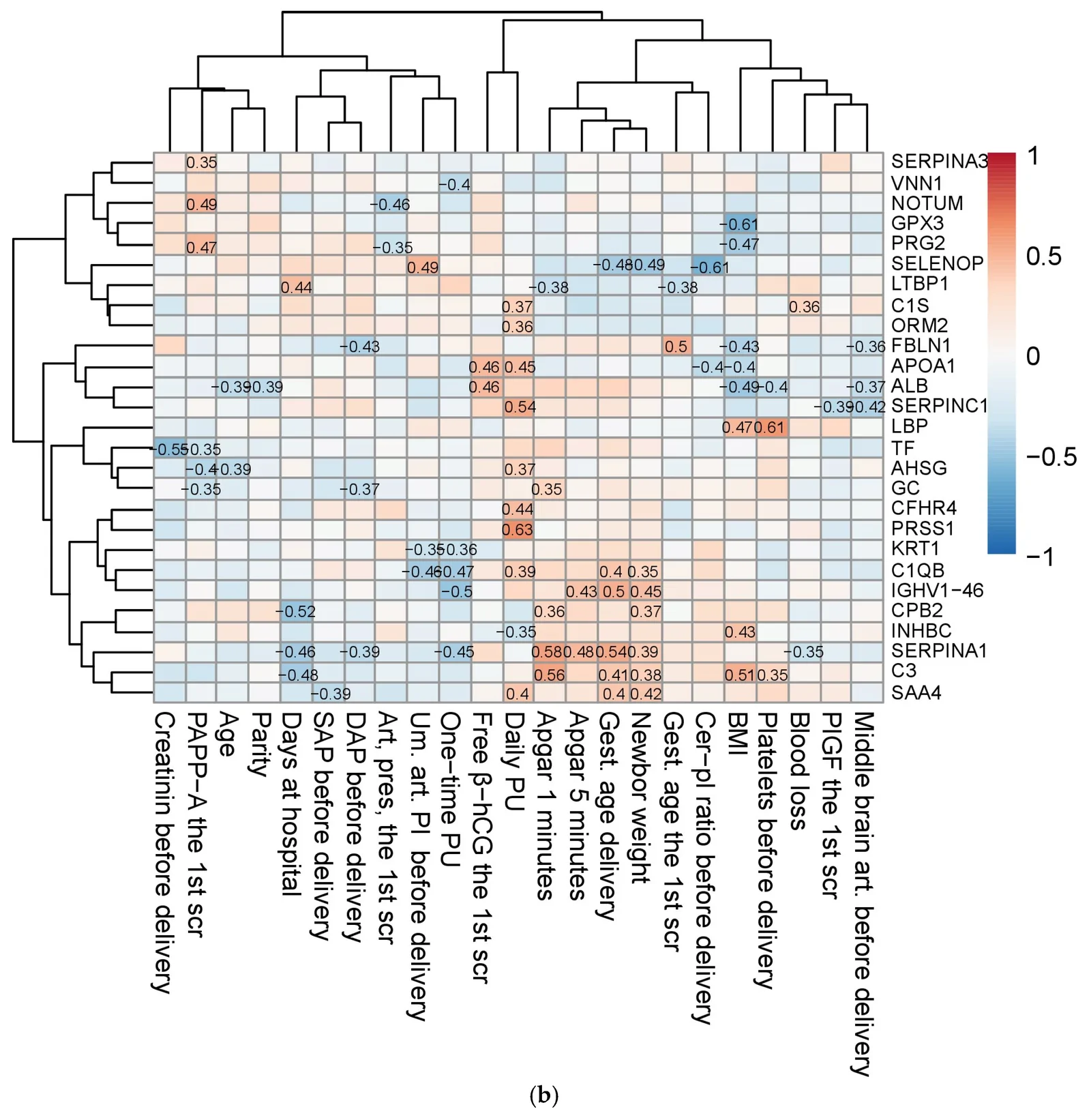
Integrated correlation analysis of proteomic markers and clinical characteristics for PE: heatmaps of statistically significant associations (*p* < 0.05) between key variables. Cells with non-zero correlation coefficients are color-coded according to the strength and direction of the correlation (red for positive, blue for negative). Each significant cell is annotated with the exact correlation coefficient value (e.g., *r* = 0.45) to facilitate precise interpretation. (**a**) Correlation heatmap between DIA-MS markers (rows) and MRM-MS markers (columns) of PE. This panel highlights the concordance and complementarity between protein markers identified via semi-quantitative, non-targeted DIA-MS and quantitative, targeted MRM-MS. Significant correlations suggest technical reproducibility and biological consistency across the two proteomic platforms. (**b**) Correlation heatmap between DIA-MS markers of PE and clinical characteristics of the PE group (*n* = 32). Statistically significant correlations indicate potential links between molecular signatures and clinically measurable phenotypes, supporting the biological relevance of the identified markers.

Correlation analysis between DIA-MS and MRM-MS measurements demonstrated significant concordance for the majority of overlapping proteins: 71% showed Pearson correlation coefficients greater than 0.5 with *p* < 0.001, indicating good reproducibility between the semi-quantitative and quantitative approaches (Table S4). A correlation heatmap highlighted four coherent clusters of markers across platforms, including a tight subcluster comprising AHSG, C8A and F10 from the MRM panel and AHSG, C1QB and PRSS1 from the DIA panel, all with correlation coefficients above 0.6. *AHSG* levels measured by DIA-MS and MRM-MS were particularly consistent, with a correlation coefficient of 0.82 (*p* < 0.001), supporting its role as a robust early marker candidate (Figure 6a).

### 3.4. Associations Between Protein Markers and Clinical Parameters

Linear mixed models were used to assess whether clinical parameters with significant group differences (nulliparity, previous PE, habitual miscarriage, previous preterm delivery, etc.; Table 1) confounded protein marker levels. None of the demographic or clinical covariates showed statistically significant confounding effects on DIA-MS protein markers (*p* < 0.05, Table S5). This indicates that the observed proteomic differences between PE cases and controls are not merely surrogates for these clinical risk factors, but rather reflect independent biological signals associated with disease development.

Integration of DIA-MS protein markers with clinical data revealed two main clusters of clinical variables on the correlation heatmap (Figure 4b). Gestational age at delivery, birthweight, and Apgar scores at 1 and 5 min formed a cluster of positive associations with several proteins from the DIA-MS dataset, including C1QB, IGHV1-46, CPB2, SERPINA1, C3, and SAA4.

In contrast, markers of disease severity—including 24 h proteinuria, umbilical artery pulsatility index, and pre-delivery creatinine levels—formed a separate cluster, predominantly showing inverse correlations with the same protein set. Daily proteinuria exhibited a weak negative association with *CPB2* (r = −0.35) and positive associations with SAA4, C1QB, PRSS1, AHSG, CFHR4, SERPINC1, APOA1, ORM2, and C1S. The strongest positive correlations with daily proteinuria were observed for PRSS1 (r = 0.63) and SERPINC1 (r = 0.54). Spot proteinuria showed negative associations with SERPINA1, IGHV1-46, C1QB, KRT1, and VNN1.

Additionally, platelet count demonstrated a strong positive association with LBP (r = 0.61), while the cerebroplacental ratio (CPR) showed a strong negative association with SELENOP (r = −0.61).

## 4. Discussion

The FMF algorithm, despite its established clinical track record, demonstrates variable performance, detecting 80.6% (95% CI 64.0–91.8) of preterm PE cases (<37 weeks) and only 31.8% (95% CI 18.6–47.6) of term PE cases (≥37 weeks) [55]. Moreover, false-negative results persist in real-world practice. Some women without pronounced anamnestic risk factors who are classified as low risk subsequently develop severe PE, leading to adverse outcomes [3,4,11,12,55,56,57]. This may be attributed to operator dependence in UtA-PI measurement, insufficient sphygmomanometer quality control, and lack of regular calibration. In the present cohort, the FMF model identified high risk in only 53% of women who later developed PE, underscoring the substantial clinical need for a more sensitive and reproducible screening approach.

Integrated untargeted DIA-MS and targeted MRM-MS profiling (*n* = 64) of first-trimester maternal serum reliably identified women at risk for subsequent PE. The discovery phase yielded 33 DIA-MS markers (FDR < 0.01, VIP > 1.5), achieving excellent predictive performance (AUC 0.95, sensitivity 95%, specificity 97%) using SVM and random forest classifiers. Targeted MRM-MS verified nine proteins, of which four—AFM, AHSG, C8A, and IGHG1—confirmed the discovery findings. High cross-platform concordance (71% of overlapping proteins with r > 0.5, *p* < 0.001) underscores technical reproducibility. 

A direct head-to-head comparison revealed that the SVM model based on the protein signature significantly outperformed the FMF model, achieving a superior AUC (0.95 vs. 0.77) and notably higher sensitivity (94% vs. 53%). This represents a marked improvement in the detection rate for future PE cases, with the proteomic model identifying nearly twice as many at-risk women. Notably, the addition of clinical and anamnestic parameters to the proteomic model did not further enhance predictive performance, suggesting that the protein signature captures the underlying biological state associated with these conventional risk factors. The proteomic model demonstrated excellent calibration (ECE = 0.047) and superior net benefit across a range of clinically relevant decision thresholds, as shown by decision curve analysis. Furthermore, the significant NRI (0.38; 95% CI: 0.20–0.56) indicates that incorporating this proteomic panel would lead to more accurate risk reclassification, potentially sparing low-risk women unnecessary interventions while directing high-risk women toward timely prophylaxis and enhanced surveillance.

These findings are consistent with previous first-trimester proteomic studies [15,23,58,59], collectively demonstrating that serum proteomic signatures can detect early molecular changes preceding clinical disease. This capability is particularly critical given the limited effectiveness of current prevention strategies: low-dose aspirin reduces PE risk by only 18–48% [56,57]. This modest efficacy likely reflects biological heterogeneity, encompassing distinct molecular subclasses such as placental, metabolic, maternal anti-fetal, and extracellular matrix-related subtypes [16,60,61]. A proteomic approach that captures this heterogeneity holds promise for more precise risk stratification and ultimately for enabling personalized preventive strategies tailored to each patient’s underlying pathophysiology.

The biological coherence of the discovered markers strengthens their clinical plausibility. Pathway enrichment analysis prominently featured complement cascade activation, a central mechanism in PE pathogenesis [16,60,62,63]. Altered concentrations of complement-related proteins, including C1QB, C3, CFHR4, and other components of the classical pathway, may reflect their consumption due to early complement activation. Several complement-related proteins, including C1QB, C1S, C3, C8A, and C9, were significantly altered in women who later developed PE, consistent with a chronic low-grade inflammatory and complement-dysregulated state established in the first trimester [15,62,63,64,65]. Interestingly, a prospective study by He YD et al. (2020) demonstrated that by the second and third trimesters, blood profiles differ from those in women with uncomplicated pregnancies [62]. Furthermore, Matsuyama T. et al. (2021) showed in vitro that an imbalance of pro-angiogenic and anti-angiogenic factors—observed as early as the first trimester in pregnancies subsequently complicated by PE—inhibits the synthesis of complement factor H by placental endothelial cells, leading to complement activation and endothelial dysfunction [63].

Similarly, several liver-derived and transport proteins—such as albumin (ALB), alpha-1-antitrypsin (SERPINA1), alpha-1-antichymotrypsin (SERPINA3), serotransferrin (*TF*), and vitamin D-binding protein (*GC*)—may reflect early changes in hepatic synthetic function, systemic inflammation, or redistribution processes previously associated with adverse pregnancy outcomes [15,23]. Reduced first-trimester ALB levels have demonstrated moderate prognostic value for PE; however, its diagnostic performance improves substantially when incorporated into combined predictive models [15,23]. SERPINA1 has been proposed as a potential component of proteomic panels for early PE prediction, demonstrating high diagnostic accuracy within multi-protein models [23]. Experimental evidence indicates its involvement in regulating trophoblast invasion through endoplasmic reticulum stress pathways [66]. Elevated levels of non-tryptic SERPINA1 peptides in urine are associated with the clinical course of preeclampsia and may represent a promising non-invasive marker of disease severity [64,67,68]. By contrast, SERPINA3—belonging to the same family of serine protease inhibitors—has not shown a significant association between genetic variants and PE risk [69], and its role in disease pathogenesis remains incompletely understood.

Thus, early disturbances in hepatic synthetic and metabolic function may play an important role in the pathogenesis of preeclampsia as early as the first trimester. Elevated first-trimester hepatic steatosis index (HSI) values were recently associated with an increased risk of gestational hypertension and PE in a study by Zhang et al. (2025) [70], confirming the role of early structural liver changes closely linked to metabolic and functional dysfunction. The downregulation of AHSG—a liver-synthesized protein involved in metabolic regulation—observed in both DIA-MS and MRM-MS platforms (Pearson r = 0.82, *p* < 0.001) aligns with previous reports linking low AHSG to preterm PE and to metabolic dysfunction-associated steatotic liver disease (MASLD) [71], which itself associates with adverse pregnancy outcomes [72,73]. A longitudinal study by Chaemsaithong P. et al. (2015) found that AHSG levels increased from the first trimester to 26 weeks of gestation and then declined, being significantly lower in the group that developed early-onset PE [74]. However, the direction of AHSG changes varies across studies: both elevated levels in manifest PE and reduced levels in early-onset disease and longitudinal observations have been reported, reflecting disease heterogeneity, differences in gestational timing, and population characteristics [74,75]. Reduced levels of several proteins, including AHSG, may be attributable to multiple mechanisms: redistribution and local accumulation in placental tissue (as shown for SERPINA1) [64,76] and consumption under conditions of immune cascade activation [15,23,65], as well as differences in disease stage and phenotypic heterogeneity [16,60].

Accumulating evidence points to a possible role of hemostasis imbalance in the development of PE. Pei-Pei Jin et al. (2023) [77] conducted a prospective study consistent with the present findings, evaluating first-trimester antithrombin III (SERPINC1) levels in 853 pregnant women (322 who subsequently developed PE and 531 controls). That study demonstrated that reduced SERPINC1 levels were significantly associated with disease development after adjustment for age and BMI [77]. The role of other coagulation proteins, such as PROC and CPB2, in early PE prediction remains limited; nevertheless, hemostatic disturbances confirm their involvement in disease pathogenesis [65]. Furthermore, the downregulation of GPX3, SERPINC1, and SERPINA1 points to impaired antioxidant capacity and coagulation regulation, which are both implicated in PE progression [66,69,75,76,77].

Proteins involved in extracellular matrix (ECM) organization and vascular remodeling, including fibulin-1 (FBLN1), may reflect impaired vascular adaptation and defective placentation. This aligns with evidence implicating ECM dysregulation in PE pathogenesis [78]. First-trimester proteomic studies have demonstrated the potential of ECM-related proteins as early disease biomarkers, and experimental data indicate that alterations in collagen composition may disrupt trophoblast function and contribute to defective placentation [23,78]. 

An important translational insight emerges from the correlation analysis between protein markers and clinical parameters (Figure 4b). PRSS1, IGHV1-4, and SERPINC1 showed a strong correlation with proteinuria (|r| > 0.5), directly linking the proteomic signature to clinically meaningful endophenotypes of disease severity. Moreover, the associations of IGHV1-46, AHSG, SERPINA1, SERPINC1, C1QB and C3 with umbilical artery pulsatility indices, gestational age at delivery and duration of hospitalization suggest that these markers capture not only the risk of developing PE but also the anticipated trajectory of placental and maternal organ dysfunction. This dual prognostic capacity—predicting both the occurrence and the likely severity of PE—represents an advance over many existing single-analyte or purely demographic models [3,63,75].

The proteomic findings of this study must be considered within the broader context of emerging environmental and biological contributors to PE pathogenesis. Beyond classical pathways of complement activation, coagulation, and hepatic metabolism, recent evidence implicates microplastics, endocrine-disrupting chemicals (EDCs), such as BPA and phthalates, air pollution, gut microbiota dysbiosis, oxidative stress, and chronic low-grade inflammation in abnormal placentation and endothelial dysfunction [79,80,81,82]. Microplastics and EDCs have been detected in human placenta and can impair trophoblast invasion via *IL-6/STAT3* signaling and placental microRNA dysregulation [79,83,84,85]. Airborne particulate matter induces trophoblast oxidative stress through the KLF9/CYP1A1 pathway, while gut dysbiosis promotes inflammatory responses via the “SCFAs–cathepsin C–macrophage polarization” axis and FMO3-driven inflammation [80,86]. Oxidative stress serves as a central unifying mechanism, with BPA activating ROS-driven apoptotic pathways, phthalates depleting glutathione stores, and air pollution triggering NLRP3 inflammasome activation [81,82,87].

The proteomic data presented here directly support several of these pathways: downregulation of GPX3 points to impaired antioxidant defense, altered complement proteins (C1QB, C3, C8A, CFHR4) reflect chronic inflammation, and changes in SERPINC1 and SERPINA1 align with hemostatic and inflammatory disturbances potentially exacerbated by environmental toxins [60,69,73]. While direct proteomic evidence linking these emerging mechanisms to specific markers remains limited, the convergence of these findings with the broader literature strengthens the biological plausibility of the identified signature [15,23]. Future research should investigate whether environmental exposure burden or gut microbial composition influences the predictive performance of this proteomic panel and its association with distinct molecular subclasses of PE [16,60,61].

From a methodological perspective, the combination of DIA-PASEF-MS for broad discovery and MRM-MS for multiplexed, absolute quantification proved highly synergistic. The DIA-MS platform enabled unbiased interrogation of >450 protein groups in blood serum, while the targeted MRM panel—originally designed for other disease contexts but applied here to PE—permitted rapid, quantitative orthogonal technical confirmation of candidate markers without requiring de novo assay development [18,20,21,22,23,24,26]. The high cross-platform correlation for more than 70% of overlapping proteins indicates that semi-quantitative DIA-MS data can be reliably used for initial screening, with MRM-MS serving as a confirmatory or clinical-grade quantification method. Of particular interest is the identification of coherent protein clusters demonstrating similar patterns of change across both methods. Specifically, a tightly connected subcluster was identified, including AHSG, C8A, and F10 (by MRM-MS), as well as AHSG, C1QB, and PRSS1 (by DIA-MS), all of which exhibited high correlation coefficients. This indicates coordinated changes in these proteins and likely reflects their involvement in common pathophysiological processes, such as complement activation, inflammatory response, and hemostatic disturbances. The highest cross-platform concordance was observed for AHSG, confirming its robustness to analytical variability and positioning it as one of the most reliable candidate early biomarkers of preeclampsia.

Several limitations warrant consideration. First, the current findings should be interpreted as hypothesis-generating and exploratory. The sample size (*n* = 64), while adequately powered for discovery and for training machine learning models with cross-validation, requires external validation in larger, multi-center cohorts before clinical implementation. Second, the study was conducted at a single tertiary referral center with a predominantly nulliparous and Caucasian population; generalizability to other ethnic groups, multiparous women, and lower-resource settings remains to be established. We strongly caution against the extrapolation of these results as a race-specific diagnostic marker without validation in larger, multi-ethnic populations.

Third, the control group selection strategy—whereby controls were required to have both uncomplicated pregnancies and no high FMF-based first-trimester PE risk—may have artificially enhanced the clinical separation between cases and controls. While this design was chosen to minimize the inclusion of controls with subclinical disease or latent risk factors, it precludes a direct assessment of the incremental value of proteomics specifically over and above the FMF model in a general screening population. However, the head-to-head comparison and NRI analysis performed in this study (AUC 0.95 vs. 0.77, NRI 0.38) indicate that the proteomic signature still provides substantial added predictive value, even in this enriched cohort.

Fourth, the MRM panel, though multiplexed, was not specifically optimized for PE; a purpose-built PE panel might yield even higher performance. Fifth, proteomic analysis was performed at a single time point in the first trimester, which does not allow assessment of the dynamics of molecular changes throughout pregnancy. Longitudinal sampling—particularly in the second and third trimesters—would provide valuable insights into the temporal evolution of the proteomic signature, its association with clinical deterioration, and its potential utility for monitoring disease progression or response to preventive interventions such as aspirin. 

Sixth, while association with clinical severity parameters was assessed, the study was not designed to distinguish early- vs. late-onset PE or preterm vs. term PE, which may represent biologically distinct entities with distinct proteomic profiles [15,16,60,61]. A subgroup analysis comparing early-onset (*n* = 8) and late-onset (*n* = 24) PE cases revealed no statistically significant differences in first-trimester serum protein expression, with PCA showing poor separation between groups and an OPLS-DA model exhibiting limited predictive capacity (Q^2^Y = 0.18), likely attributable to the substantial class imbalance (ratio of 3:1). Consequently, a larger, adequately powered study with balanced representation of early- and late-onset PE cases is required to robustly investigate subtype-specific biomarkers and to determine whether the identified proteomic signature is preferentially associated with a particular disease subtype.

Despite these limitations, the present findings align with and extend the growing body of literature implicating complement, coagulation, and metabolic regulation pathways in early PE pathogenesis [15,16,58]. The four cross-platform verified markers (AFM, AHSG, C8A, IGHG1) represent particularly robust candidates for further development. The ability to predict PE from first-trimester serum using quantitative MS—without requiring specialized ultrasonographic equipment or operator training—suggests potential utility, particularly in settings where Doppler expertise is limited, should this approach be validated in future studies. The high NPV (95%) observed in this cohort, while encouraging, requires confirmation in external validation studies before any clinical reassurance can be offered.

## 5. Conclusions

This study demonstrates that integrated untargeted DIA-MS and targeted MRM-MS profiling of first-trimester maternal serum can identify women at risk for subsequent preeclampsia. In a discovery cohort (*n* = 64), the analysis identified 33 DIA-MS markers (FDR < 0.01, VIP > 1.5), with four proteins (AFM, AHSG, C8A, IGHG1) confirmed by cross-platform MRM-MS analysis. The SVM and random forest classifiers achieved promising predictive performance (AUC 0.95, sensitivity 94%, specificity 97%) in internal cross-validation, and the proteomic signature demonstrated superior performance compared to the FMF screening algorithm (AUC 0.95 vs. 0.77). High cross-platform concordance (71% of overlapping proteins with r > 0.5, *p* < 0.001) underscores technical reproducibility.

Pathway enrichment featured complement activation, IGF transport dysregulation, and platelet degranulation. PRSS1, IGHV1-4, and SERPINC1 showed a strong correlation with proteinuria (|r| > 0.5), directly linking the proteomic signature to clinically meaningful manifestations of disease severity. The four cross-platform validated markers represent robust candidates for further development. 

The ability to predict PE from first-trimester serum using quantitative mass spectrometry—without specialized ultrasound equipment or operator training—suggests potential utility in settings where Doppler expertise is limited. However, the study was conducted in a single center with a predominantly nulliparous Caucasian population, and the model was developed and validated using internal cross-validation. Therefore, the current findings should be interpreted as hypothesis-generating and proof-of-concept. Independent validation in larger, prospective, multi-center cohorts with diverse ethnic and demographic populations is essential before any clinical implementation can be considered. The high negative predictive value (95%) observed in this cohort, while encouraging, requires confirmation in external validation studies.

Future directions include external validation in diverse populations, prospective comparison with the FMF model, development of a clinically validated targeted panel, and investigation of whether the proteomic signature can guide aspirin prophylaxis or other preventive strategies. If successfully validated, integrating quantitative proteomics into first-trimester screening may improve early identification of women at risk for PE, enabling timely surveillance and preventive therapy. For now, this approach represents a promising step toward more personalized risk stratification that warrants further investigation.

## Figures and Tables

**Table 1:** Clinical characteristics of the control and PE groups. Continuous data are presented as median (Q1; Q3); categorical data are presented as *n* (%). *p*-values were calculated using the Mann–Whitney U test for continuous variables and Pearson’s chi-square test for categorical variables. BMI—body mass index, IVF—in vitro fertilization, MAP—mean arterial pressure, SBP—systolic blood pressure, DBP—diastolic blood pressure, PlGF—placental growth factor, UA-PI—umbilical artery pulsatility index, CPR—cerebroplacental ratio, CS—caesarian section.

Feature	Control (*n* = 32)	PE (*n* = 32)	*p*-Value
Age, years, Me[Q1;Q3]	31.6 (29.4; 34.75)	33.35 (29.08; 37.45)	0.08
BMI, Me[Q1;Q3]	21.49 (19.72; 22.89)	22.74 (20.36; 24.52)	0.07
Previous PE, *n* (%)	0(0%)	7(22%)	0.01
Nulliparous, *n* (%)	11(34%)	24(72%)	<0.001
IVF, *n* (%)	0(0%)	2(6%)	0.49
Habitual miscarriage, *n* (%)	0(0%)	12(38%)	<0.001
Previous preterm delivery, *n* (%)	0(0%)	8(25%)	0.005
Gestational age at sample collection, wks, Me[Q1;Q3]	12.14 (11.86; 12.93)	12.29 (12.11; 12.57)	0.73
MAP, MoM, Me[Q1;Q3]	1 (0.95; 1.04)	1.04 (0.97; 1.12)	0.1
PIGF (1st trimester prenatal screening), MoM, Me[Q1;Q3]	0.74 (0.54; 1.06)	0.55 (0.36; 0.69)	0.002
FMF first-trimester high PE risk, *n* (%)	0(0%)	17(53%)	<0.001
Max. SBP, Me[Q1;Q3]	115 (110; 120)	135 (125; 149.75)	<0.001
Max. DBP, Me[Q1;Q3]	70 (70; 74.5)	89 (80; 99.25)	<0.001
UA-PI, Me[Q1;Q3]	0.79 (0.73; 0.88)	0.95 (0.82; 1.13)	0.002
CPR, Me[Q1;Q3]	1.89 (1.58; 2.2)	1.44 (1.31; 1.75)	<0.001
24 h proteinuria, g/L, Me[Q1;Q3]	0 (0; 0)	1.1 (0.61; 2.18)	<0.001
Creatinine, µM/L, Me[Q1;Q3]	66.6 (63.3; 69.45)	80.8 (70.85; 87.77)	<0.001
Gestational age at delivery, wks, Me[Q1;Q3]	39.4 (38.55; 40)	37.2 (34.8; 38)	<0.001
Blood loss at delivery, mL, Me[Q1;Q3]	300 (250; 350)	700 (475; 700)	<0.001
Emergency CS, *n* (%)	1(3%)	19(59%)	<0.001
Apgar score at 1 min	8 (8; 8)	8 (7; 8)	0.002
Apgar score at 5 min	9 (9; 9)	8.5 (8; 9)	0.001
Newborn weight, g, Me[Q1;Q3]	3400 (3215; 3613)	2745 (1691; 3097.5)	<0.001

**Table 2:** Quality metrics of machine learning models for predicting PE from first-trimester serum protein markers detected by DIA-MS coupled with clinical and anamnestic parameters. Models included OPLS-DA, SVM (linear, polynomial, radial and sigmoid kernels) with PSO-optimized hyperparameters and random forest. Performance was evaluated by 10-fold cross-validation, reporting sensitivity, specificity, accuracy, AUC, PPV, NPV, and F-score with confidence interval.

Model	Accuracy, %	Sensitivity, %	Specificity, %	AUC	PPV	NPV	F-Score
OPLS-DA	95% (89–100%)	94% (84–100%)	97% (91–100%)	0.95 (0.91–1)	0.97 (0.91–1)	0.94 (0.86–1)	0.95 (0.87–1)
SVM, linear kernel	95% (89–100%)	94% (84–100%)	97% (91–100%)	0.95 (0.91–1)	0.97 (0.91–1)	0.94 (0.86–1)	0.95 (0.87–1)
SVM, polynomial kernel	95% (89–100%)	94% (84–100%)	97% (91–100%)	0.95 (0.91–1)	0.97 (0.91–1)	0.94 (0.86–1)	0.95 (0.87–1)
SVM., radial kernel	95% (89–100%)	94% (84–100%)	97% (91–100%)	0.95 (0.91–1)	0.97 (0.91–1)	0.94 (0.86–1)	0.95 (0.87–1)
SVM, sigmoid kernel	91% (84–97%)	88% (75–97%)	94% (84–100%)	0.91 (0.83–0.97)	0.93 (0.84–1)	0.88 (0.79–0.97)	0.90 (0.79–0.98)
Random Forest	95% (89–100%)	94% (84–100%)	97% (91–100%)	0.95 (0.91–1)	0.97 (0.91–1)	0.94 (0.86–1)	0.95 (0.87–1)

**Table 3:** Comparative performance of the best proteomic model, the combined proteomic–clinical model, and the FMF screening algorithm. Expected calibration error (ECE) is provided to assess model calibration.

Model	Accuracy	Sensitivity	Specificity	AUC	ECE
FMF	76% (69–86%)	53% (38–72%)	100% (100–100%)	0.77 (0.68–0.85)	0.24
Proteomic model	95% (89–100%)	94% (84–100%)	97% (91–100%)	0.95 (0.91–1)	0.047
Proteomic + clinical model	95% (89–100%)	94% (84–100%)	97% (91–100%)	0.95 (0.91–1)	0.047

## Data Availability

Data are available via ProteomeXchange with identifier PXD080843.

## Supplementary Materials

The following supporting information can be downloaded at https://www.mdpi.com/article/10.3390/life16081264/s1, Table S1. DIA-MS–quantified protein levels in control (*n* = 32) and PE (*n* = 32) groups: descriptive statistics, fold change, statistical significance, adjusted *p*-values by Benjamini-Hochberg correction, test power, and VIP from OPLS-DA Model; Table S2. Statistically significant enriched pathways identified from DIA-MS protein markers of preeclampsia (PE). Pathway enrichment analysis was performed using the STRING database with a false discovery rate (FDR) threshold of < 0.01. The analysis revealed that 33 DIA-derived PE protein markers were significantly enriched in complement activation pathways. Additionally, these markers were associated with several key biological processes, including post-translational protein phosphorylation, regulation of insulin-like growth factor (IGF) transport and uptake, platelet degranulation, and selenium-related micronutrient networks; Table S3. Levels of proteins measured by MRM-MS in the control (*n* = 32) and PE (*n* = 32) groups. Data are presented as median (first quartile; third quartile). For each protein, the table includes fold change, statistical significance (*p*-value), Benjamini–Hochberg adjusted *p*-value (p-adj), statistical power, and variable importance projection (VIP) score derived from the OPLS-DA model; Table S4. Correlation between serum protein levels measured by quantitative MRM-MS and semi-quantitative DIA-MS. Associations between DIA-MS and MRM-MS protein levels were assessed using Pearson correlation. For each protein, the table reports the correlation coefficient, its 95% confidence interval (CI), and the corresponding statistical significance (*p*-value); Table S5. Coefficients and statistical significant levels of the coefficients of potential cofounder of PE in linear-mixed models of protein markers levels.

## Abbreviations

The following abbreviations are used in this manuscript:

AUC	Area under receive operational curve
BMI	Body mass index
MAP	Mean arterial pressure
SBP	Systolic blood pressure
DBP	Diastolic blood pressure
DIA	Data-independent acquisition
FDR	False discovery rate
HPLC	High-performance liquid chromatography
LLOQ	Lower limit of quantification
MoM	Multiples of medians
MRM	Multiple reaction monitoring
MS	Mass spectrometry
NAT	Natural synthetic proteotypic peptides
OPLS-DA	Orthogonal projection on latent structures discriminant analysis
QC	Quality control
PCA	Principal component analysis
PE	Preeclampsia
CS	Caesarian section
CPR	Cerebroplacental ratio
UtA-PI	Uterine artery pulsatility index
UA-PI	Umbilical artery pulsatility index
PlGF	Placental growth factor
ROC	Receiver operating characteristic
SIS	Stable isotope labeled standard
SVM	Support vector machine
VIP	Variable importance in projection
ECE	Expected calibration error
NRI	Net reclassification improvement
DIA-PASEF	Data-Independent Acquisition—Parallel Accumulation Serial Fragmentation
